# Corporate Medical Lead

**Published:** 1920-04-10

**Authors:** 


THE PUBLIC WELL-BEING—(*continued*).

### Corporate Medical Lead.

HAVING written with some freedom on the American criticism of some English methods, let us now, so to say, offer the other cheek by drawing attention to the annual report of the Public Health Committee of the New York Academy of Medicine for the year 1919. The objects and work of this Committee are to furnish authoritative and unbiased opinions on public health, hospital management, and medical education, and to initiate and supervise activities coming under these heads. It is able to do this work because it receives the assistance of the expert knowledge which exists in the Academy of Medicine. It deals with all forms of disease and with health administration, organises courses for health officers, makes public knowledge of preventive measures against certain diseases, gives a lead to local public authorities in such things as organisation of a school lunch system, early treatment of industrial neuroses, and generally keeps an eye on the administration of the city and State.

Among the subjects which have definitely come within the activities of the Committee during the year are convalescent treatment in hospital, reorganisation of the City Department of Health, medical examination of immigrants, medical education, the mosquito nuisance in New York, the milk question, daylight saving, health centres, etc. The Committee is obviously carrying on a valuable consultative and constructive work. In this country it might be quite useful to have similar kind of Health Committees, composed of medical men, in the various towns and centres. At the top the Ministry of Health already has something very similar to the Committee of the New York Academy of Medicine in its Consultative Council, which embraces many of the leading medical men of the land. Such Committees are not so vitally necessary where there is a Health Ministry containing enlightened and progressive men such as ours, but there seems a great deal to be said for creating not only local Committees for taking a detached provisional view and lead on local questions, but also an authoritative Committee of the whole medical profession, which would speak with one voice on the various problems that arise from time to time concerning public health and administration.

